# Trench Fever Investigations

**Published:** 1918-12

**Authors:** 


### TRENCH FEVER INVESTIGATIONS

The Trench Fever Commission of the American Red Cross Research Committee, consisting of Lt.-Colonel Richard P. Strong, Major Homer F. Swift, Major Eugene L. Opie, Major J. Ward MacNeal, Captain Walter Baetjer, Captain A. M. Pappenheimer, Captain A. D. Peacock, and Captain David Rapport, operating in co-operation with the medical departments of the British Armies in France and the American Expeditionary Forces, has completed its work. The thoroughness with which the investigations were carried out, before the Trench Fever Commission was willing to announce its conclusions, has not been excelled in the history of medical research, and the importance of its findings has been farreaching in solving one of the great sanitary problems of the war.

Preliminary statements by the Commission, announcing the results of the experiments which have proved that the louse transmits trench fever, and also the reports on other studies of various phases of the disease, have been given out from time to time. The Commission having completed its work, the American Red Cross in France has had its report published by the Oxford University Press, and it is ready for distribution.

### A Medical Classic.

The Commission's monograph on trench fever makes a book of absorbing interest and of very great scientific value. Indeed, it is one of the most important literary contributions of the war; and although called a " Report ", it is really a treatise on trench fever that will take its place among the classics of medical literature.

The typographical and general make-up of this book is of the highest order, and, in printing this attractive medical work under war conditions, the Oxford University Press demonstrates a remarkable degree of efficiency in book publication. It should be known to American medical officers that when the Bureau of Publications of the American Red Cross in France undertook to publish the Commission's book on trench fever, it was found that the paper needed for it could not be procured at any price in France. It was important to get the book out as early as possible, and there were many difficulties and delays in getting paper from the United States. Therefore, an effort was made to purchase the paper in London, but without success. Then Sir William Osler, who has the habit of accomplishing the seemingly impossible, was appealed to, and it required not more than three minutes for him to arrange with the Oxford University Press to publish the book within thirty days.

It is impossible within the limits of an editorial review to outline the investigations pursued, and the conclusions reached, by the Trench Fever Commission. The authors, after discussing the history of trench fever, give perhaps the best description that has yet appeared of the symptoms and differential diagnosis of the disease. The description of the inoculation of trench fever, and of the experiments carried out by the Commission, resulting in the proof of its transmission by the pediculus humanus, are particularly well expressed; and the case reports of the eighty-one American soldiers, who voluntarily submitted to these experiments, add greatly to the value and interest of the work. This treatise on trench fever not only gives an account of the work of the Commission, but it contains all of the most important facts regarding the disease that were known at the time of its publication.

### Conclusions Regarding the Etiology of Trench Fever.

The Commission summarizes the important conclusions drawn from its investigations as follows:

1. That trench fever is a specific, infectious disease; that it is not a modified form of typhoid or paratyphoid fever, and is not related, from an etiological standpoint, to these diseases.

2. That the organism causing the disease is a resistant filterable virus.

3. That the virus causing trench fever is present particularly in the plasms of the blood of trench fever cases, and that such plasma will produce the disease on inoculation into healthy individuals.

4. That the disease is transmitted naturally by the louse Pediculus humanus, Linn. var. Corporis, and that this is the important and common means of transmission. That the louse may transmit the disease by means of its bite alone, the usual manner of infection, or the disease may be produced artificially by scarifying the skin and rubbing in a small amount of the infected louse excrement.

5. That a man may be entirely free from lice at the time he develops trench fever, the louse that infected him having left him some time previously as its host, and that the louse need only remain upon the individual for a short period of time in order to infect him.

6. That the virus of trench fever is also sometimes present in the urine of trench fever cases, and occasionally in the sputum, and that the disease may be produced in man by the introduction of the virus in the urine or sputum through the scarified or otherwise abraded skin.

7. That since the urine and sometimes the sputum of trench fever patients are infective, these should be sterilized in order to avoid the possibility of accidental infection from them.

8. That in order to prevent trench fever or limit its spread, and thus save man-power for the armies, greater efforts must be made to keep soldiers in general from infestation with lice.

### Trench Fever Prevention

The practical application of the work is summarized in the following sanitary regulations which have been advised by the Commission:

" Exceedingly great care should be taken to completely disinfest all patients as soon as practicable, and particularly upon their entrance to the hospital. Patients on entrance should be carefully bathed, and subsequently sponged with alcohol. Their clothing and blankets should be removed, and, whether or not lice or ova are found upon them, should be carefully sterilized by moist heat at a temperature not below 70° Centigrade for half an hour, since it is possible for the virus to be still present on the clothing. It should be borne in mind that a man with trench fever may be entirely free from lice at the time that he develops symptoms of the disease. Trench fever patients should at all times be carefully protected from louse infestation, and inspection of them for lice should be made daily. They should be treated in separate wards. As the urine contains the virus and is infective, it should be sterilized during the active stages of the disease. Sputum cups should be provided for patients, and any expectorated sputum and saliva from them sterilized. Officers should regard the systematic destruction of lice as one of the most urgent of their duties, and should exercise every effort to prevent louse infestation among soldiers, and to see that any of them infected with lice are promptly disinfected and their clothing sterilized."

